# L5 Pedicle Fracture Following Single-Level L4-5 Posterior Lumbar Interbody Fusion: Successful Outcomes With Conservative Treatment in Two Cases

**DOI:** 10.7759/cureus.88197

**Published:** 2025-07-17

**Authors:** Hideki Nishi, Yukitaka Nagamoto, Tomiya Matsumoto, Takashi Kaito, Motoki Iwasaki

**Affiliations:** 1 Orthopaedics, Osaka Rosai Hospital, Sakai, JPN

**Keywords:** conservative management of spine, high l4-sagittal vertical axis (l4-sva), instrumentation failure, lumbar spine, pedicle fracture, posterior lumbar interbody fusion (plif), spinal instrumentation, teriparatide administration

## Abstract

This report describes two cases of bilateral L5 pedicle fractures following L4-5 posterior lumbar interbody fusion (PLIF) for L4 degenerative spondylolisthesis in female patients (68 and 72 years old). While this rare complication typically requires revision surgery with the extension of fixation to the sacrum or ilium, both patients achieved successful healing through conservative treatment consisting of bed rest (4-6 weeks) combined with daily teriparatide therapy (20 µg). The fractures occurred early postoperatively (5 and 36 days) and were detected through CT imaging. Both patients presented with high preoperative L4 sagittal vertical axis values (68 mm and 64 mm). At the final follow-up (36 and 18 months, respectively), both patients were free from neurological deficits with confirmed bone union. These cases represent the first report of successful conservative management of this complication. Early detection through careful monitoring in the postoperative period, particularly in cases with poor sagittal alignment of the lower lumbar spine, may lead to successful conservative treatment and help avoid revision surgery.

## Introduction

Posterior lumbar interbody fusion (PLIF) is a widely performed surgical technique for the treatment of lumbar degenerative spondylolisthesis. However, various postoperative complications following PLIF, such as adjacent segment disease, pseudarthrosis, and implant failure, are well-documented [1]. These complications have been shown to impact long-term clinical outcomes and represent major causes of revision surgery.

In contrast, pedicle fractures following PLIF represent an extremely rare complication, with limited documentation in the literature even when considering all types of posterior instrumentation surgeries [2-7]. The etiology of pedicle fractures after posterior instrumentation has been attributed to abnormal shear forces on neural arch structures [2], pedicle weakening from screw insertion [6], and mechanical stress from inadequate anterior support [4]. However, the precise pathomechanism of pedicle fractures specifically following PLIF procedures remains unclear [5].

We experienced two cases of bilateral L5 pedicle fractures following L4-5 PLIF for L4 degenerative spondylolisthesis. This complication typically requires revision surgery with extension of spinal fixation to the sacrum or ilium. However, our cases achieved successful healing through conservative treatment consisting of bed rest combined with teriparatide therapy. This report describes the clinical course of these cases and reviews the literature to elucidate the pathophysiology of this complication.

## Case presentation

**Figure 1:**
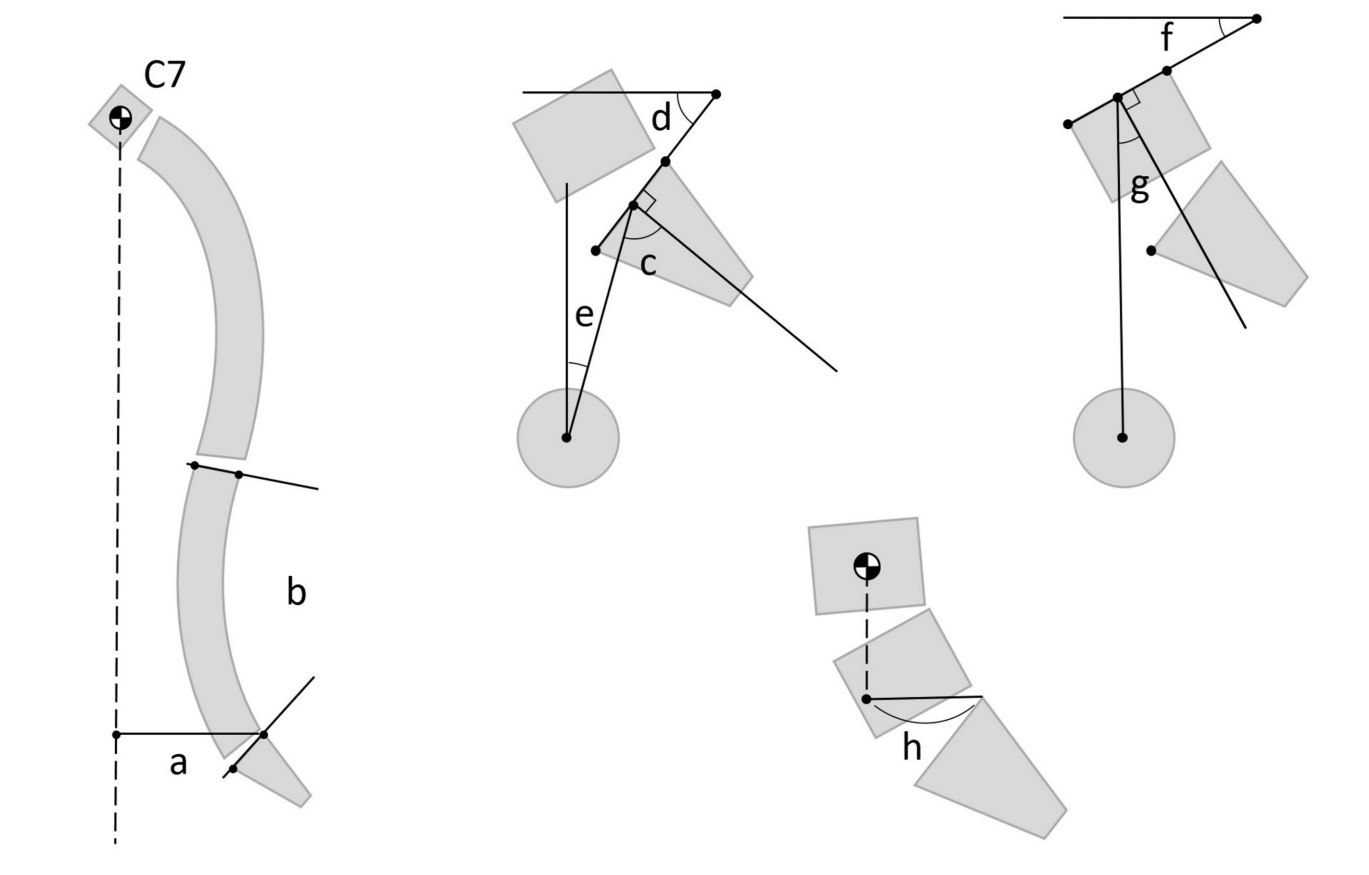
Spinal sagittal parameters a. Sagittal vertical axis (SVA): The horizontal distance between the C7 plumb line and the posterosuperior corner of S1. b. Lumbar lordosis (LL): The angle between the superior endplate of L1 and the superior endplate of S1. c. Pelvic incidence (PI): The angle between the perpendicular to the sacral plate and the line connecting the midpoint of the sacral plate. d. Sacral slope (SS): The angle between the horizontal line and the sacral plate. e. Pelvic tilt (PT): The angle between the vertical line and the line connecting the midpoint of the sacral plate to the center of the femoral heads to the center of the femoral heads. f. L5 Slope: The angle between the horizontal line and the L5 superior endplate. g. L5 Incidence: The angle between the perpendicular to the L5 superior endplate and the line connecting the midpoint of the L5 superior endplate to the center of the femoral heads. h. L4 sagittal vertical axis (L4SVA): The horizontal distance between the L4 plumb line and the posterosuperior corner of S1. Image Credits: Hideki Nishi

This study was approved by the institutional review board of Osaka Rosai Hospital, and written informed consent was obtained from both patients for the publication of this case report and accompanying images. The two cases presented in this report occurred at different times. Case 1 occurred in September 2021, while Case 2 occurred in June 2023. The treatment periods and clinical courses of the two cases were not consecutive, and each case progressed independently. Each spinal sagittal parameter used in the main text is illustrated in Figure 1.

Case 1

A 68-year-old female presented with bilateral lower limb pain and intermittent claudication for more than two years and was referred to our hospital. She had a history of hypertension and Type 1 diabetes mellitus. Preoperative dual-energy X-ray absorptiometry (DXA) scan revealed a low bone mineral density with a T-score of -3.0 and a 60% young adult mean (YAM) at the femoral neck, but the patient had no prior history of osteoporosis treatment. No neurological deficits and decreased muscle strength were observed. Plain radiographs demonstrated Meyerding grade 2 spondylolisthesis at L4-5.

**Figure 2:**
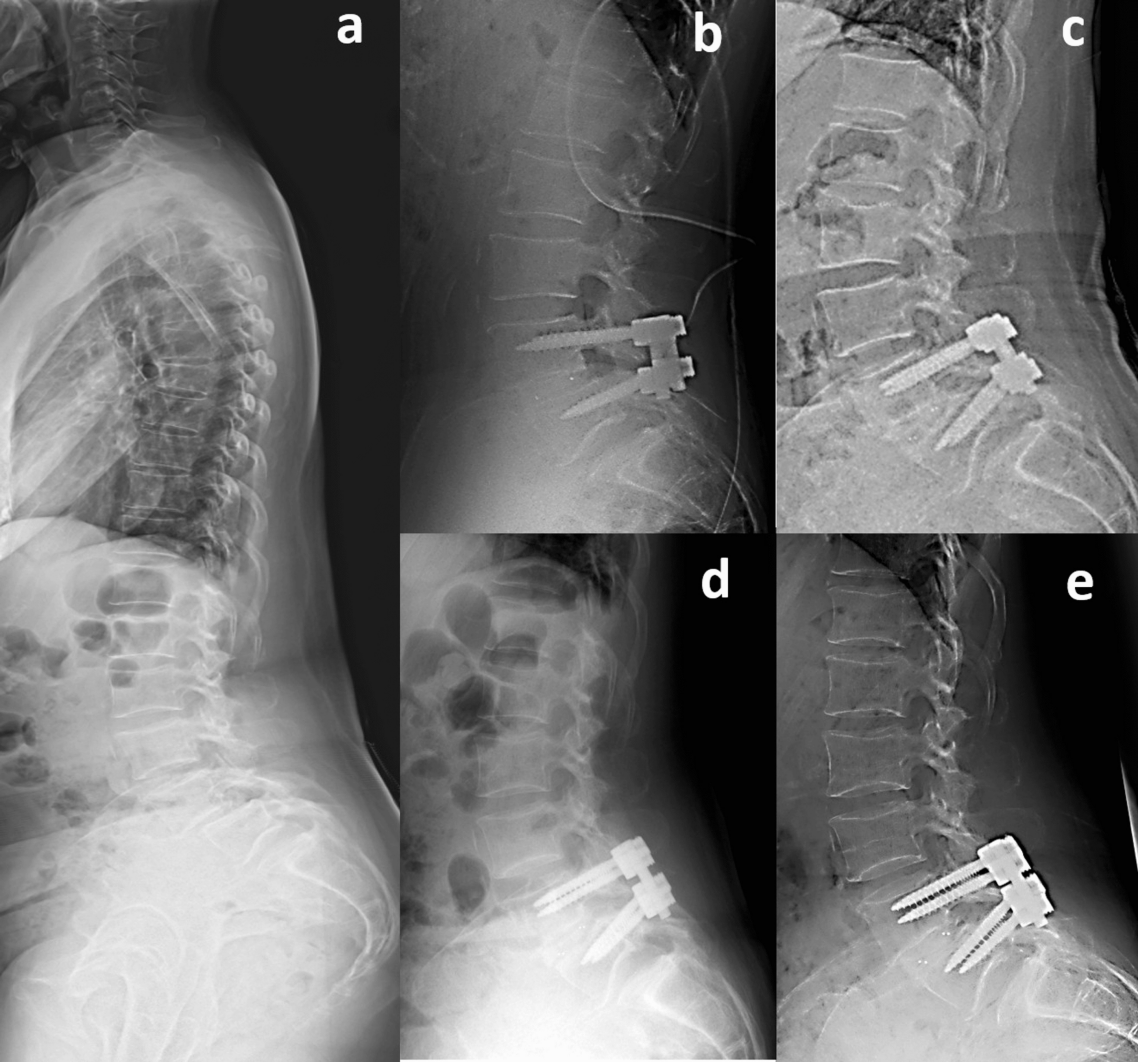
Pre and postoperative radiographs of Case 1 a. Preoperative sagittal whole-spine radiograph: lumbar lordosis (LL) 43˚, pelvic incidence (PI) 94˚, pelvic tilt (PT) 48˚, sagittal vertical axis (SVA) 108mm, L4 segmental lordosis 23 ˚ and L4-SVA 68mm. b. Immediate postoperative lateral lumbar radiograph: L4 segmental lordosis 33˚. c. Lateral lumbar radiograph at fracture onset: LL 40˚ and L4-SVA 62mm. d. Lateral lumbar radiograph after early ambulation. e. Lateral lumbar radiograph at final follow-up.

**Figure 3:**
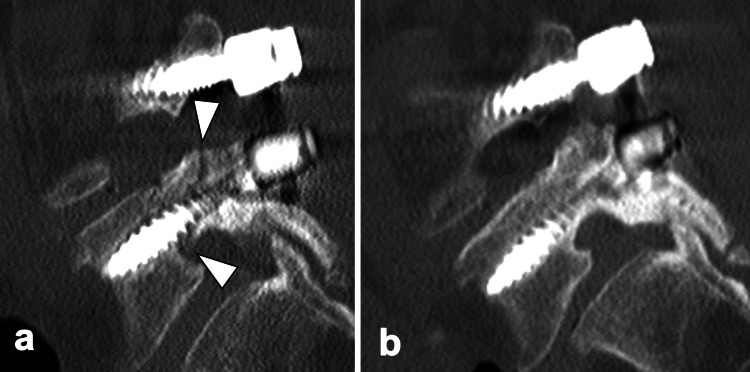
Sagittal computed tomography of the lumbar spine in Case 1 a. Sagittal computed tomography (CT) image obtained five days postoperatively demonstrating bilateral L5 pedicle fractures. b. Sagittal CT image at three years postoperatively demonstrating complete bone union of the L5 pedicle fractures.

The preoperative sagittal parameters were as follows: lumbar lordosis (LL) 43˚, pelvic incidence (PI) 94˚, pelvic tilt (PT) 48˚, sagittal vertical axis (SVA) 108 mm, and L4-SVA 68 mm (Figure 2a). Magnetic resonance imaging (MRI) demonstrated L4-5 bilateral lateral recess stenosis and right foraminal stenosis. She was diagnosed with bilateral L5 radiculopathy due to L4 degenerative spondylolisthesis and underwent PLIF at the L4-5 level. Immediate postoperative radiographs confirmed optimal implant positioning with appropriate restoration of L4-5 segmental lordosis, improving from 23° preoperatively to 33° postoperatively. In contrast, overall lumbar lordosis decreased from 43° to 33°, which was insufficient to match the patient's pelvic incidence (PI), suggesting suboptimal global sagittal alignment despite the successful local correction. Five days postoperatively, she presented with left lower limb numbness. A standing radiograph showed that L4-SVA was 62 mm (Figure 2b). Subsequent computed tomography (CT) imaging revealed bilateral L5 pedicle fractures (Figure 3a).

Initially, the extension of spinal fixation to the sacrum or ilium was considered. However, given the minimal fracture displacement, we opted for conservative treatment consisting of four weeks of bed rest combined with daily subcutaneous injections of 20 µg teriparatide. She began mobilization 4 weeks after the fracture diagnosis and was discharged home at 60 days. She used soft braces for six months postoperatively after getting out of bed. At the latest follow-up, 36 months after the procedure, the patient was completely free from neurological deficits, and bone union was obtained (Figure 3b).

Case 2

 A 72-year-old female presented with worsening right limb pain over the past several months and a recent onset of urinary frequency. She had a history of hypertension, and preoperative DXA revealed a T-score of -1.4 and 80% YAM at the femoral neck. Plain radiographs demonstrated Meyerding grade 2 spondylolisthesis at L4-5.

**Figure 4:**
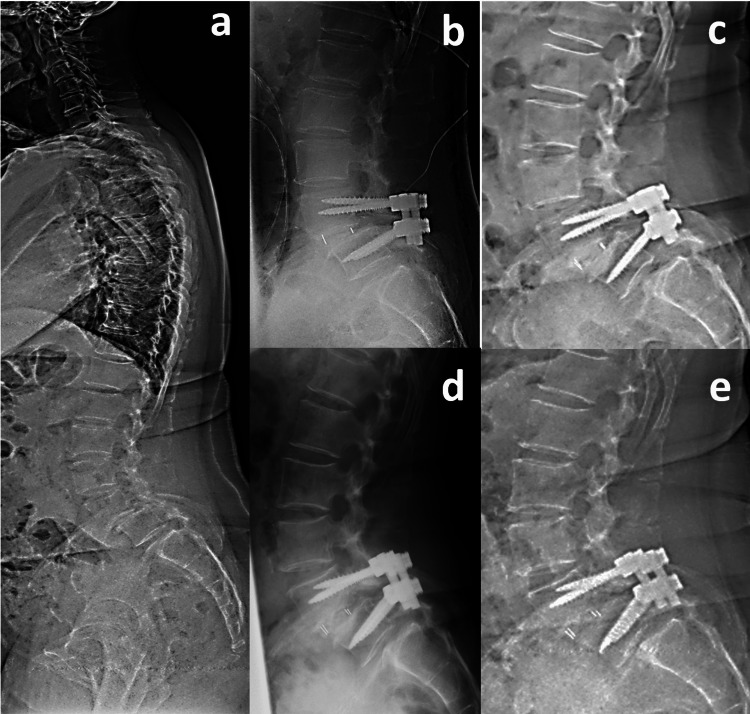
Pre and postoperative radiographs of Case 2 a. Preoperative sagittal whole-spine radiograph: LL 61˚, PI 78˚, PT 30˚, SVA 93 mm, L4 segmental lordosis 22˚, and L4-SVA 64 mm. b. Immediate postoperative lateral lumbar radiograph: L4 segmental lordosis 28˚. c. Lateral lumbar radiograph at fracture onset showing newly developed L5 spondylolisthesis: LL 60˚ and L4-SVA 65 mm. d. Lateral lumbar radiograph after early ambulation. e. Lateral lumbar radiograph at final follow-up demonstrating no further progression of spondylolisthesis.

**Figure 5:**
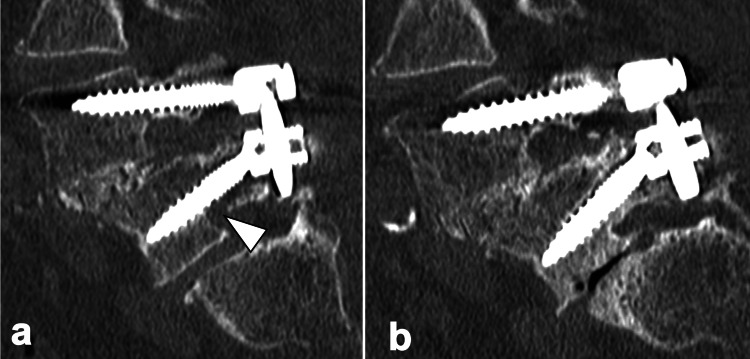
Sagittal computed tomography of the lumbar spine in Case 2 a. Sagittal CT image obtained 36 days postoperatively demonstrating bilateral L5 pedicle fractures. b. Sagittal CT image at 1.5 years postoperatively demonstrating complete bone union of the L5 pedicle fractures.

The preoperative sagittal parameters were as follows: LL 52˚, PI 78˚, PT 30˚, SVA 93 mm, and L4-SVA 64 mm (Figure 4a). MRI demonstrated L4-5 central stenosis with concurrent right foraminal stenosis. She was diagnosed with right L5 radiculopathy and cauda equina syndrome due to L4 degenerative spondylolisthesis and underwent PLIF at the L4-5 level. Immediate postoperative radiographs confirmed optimal implant positioning with appropriate restoration of L4-5 segmental lordosis, improving from 22° preoperatively to 28° postoperatively. In contrast, overall lumbar lordosis decreased from 61° to 60°, which was insufficient to match the patient's pelvic incidence (PI), suggesting suboptimal global sagittal alignment despite the successful local correction. Thirty-six days postoperatively, the patient developed lower back pain. A standing radiograph showed that L4-SVA was 65 mm (Figure 4b). Subsequent CT imaging studies revealed bilateral L5 pedicle fractures and subsequent development of L5-S1 spondylolisthesis (Figures 4c, 5a). 

In this case too, we opted for conservative treatment consisting of bed rest combined with daily subcutaneous injections of 20 µg teriparatide to avoid extending fixation to the sacrum or ilium. She began mobilization 6 weeks after the fracture diagnosis and was discharged home at 52 days. She used soft braces for six months postoperatively after getting out of bed. At the latest follow-up, 18 months after the operation, the patient was completely free from neurological deficits, and bone union was obtained (Figure 5b).

## Discussion

Pedicle fractures following lumbar posterior instrumented fusion represent a rare but potentially devastating complication, often necessitating extension of fusion to the sacrum or ilium through additional surgery [2-7]. While most previous reports have been isolated case reports encompassing diverse surgical techniques and patient demographics [2-6], pedicle stress fractures predominantly occur in long fusion constructs, with a proposed mechanism involving stress concentration at the most caudal instrumented pedicles [6]. However, cases following single-level fusion have also been documented, with fractures reported in both superior adjacent vertebrae [3,4] and caudal vertebrae similar to the present case [5]. Wanivenhaus et al. reported the first case-control study analyzing 192 cases of single-level L4-5 posterior fusion to investigate the incidence of L5 pedicle fractures and associated risk factors [7]. Their study revealed an incidence of 3.2% and identified significant risk factors, including female gender and specific lumbosacral sagittal parameters. Notably, they reported that pedicle fractures occurred exclusively in female patients in their series, which is consistent with our two cases. One of our patients demonstrated osteoporosis on preoperative dual-energy X-ray absorptiometry (DXA) scanning, suggesting that diminished bone quality may play a crucial role in the pathogenesis of this condition.

**Figure 6:**
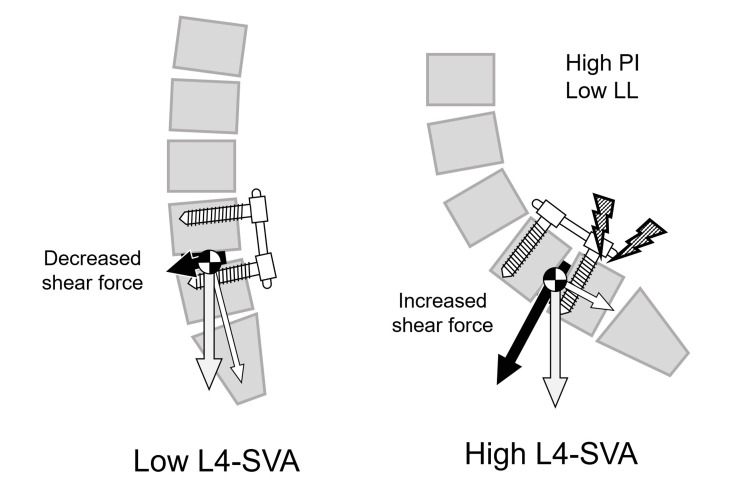
Mechanism of an L5 pedicle fracture in high L4-SVA patients In L4-5 fusion for patients with high PI and high SS, insufficient postoperative LL relative to PI and SS may lead to the anterior translation of the loading axis in the lower lumbar spine, resulting in concentrated shear forces on the L5 pedicles. The L4 sagittal vertical axis (L4-SVA), defined as the distance between the L4 plumb line and the posterosuperior corner of S1, serves as a parameter representing the anterior shift of the loading axis of the lower lumbar spine. Image Credits: Hideki Nishi

In analyzing the biomechanical basis of the fracture, Wanivenhaus et al. identified several sagittal spinopelvic parameters as risk factors, including high PI, SS, L5 slope, L5 incidence, and LL [7]. Among these parameters, all except high LL suggest the involvement of the anterior inclination of the sacral endplate in the pathophysiology of this fracture. Regarding high LL, this parameter likely developed as a compensatory response to high PI values, as evidenced by the tendency toward greater PI-LL mismatch in the fracture group (14° vs. 11° postoperatively) despite elevated LL measurements. In our cases, although we achieved satisfactory segmental lordosis at the L4-5 level through PLIF, this focal correction alone was insufficient to restore the overall LL required to compensate for the high PI values. Kitaori et al. reported a case requiring the extension of fusion to the ilium after developing the same pathology following L4-5 PLIF for L4 isthmic spondylolisthesis [5]. In explaining the pathomechanism, they cited Roussouly's analysis, which demonstrated that isthmic spondylolisthesis patients with high PI and SS develop increased shear stress at the lumbosacral junction, resulting in greater loads on the posterior neural arch [8]. Applying their theory to the present scenario, L4-5 fusion in patients with high PI and SS may result in the fusion mass tilting forward from the foundation of the sacral superior endplate if adequate postoperative LL matching their PI and SS is not achieved, consequently concentrating shear stress at the L5 pedicles at the caudal end of the fusion mass and leading to a fracture (Figure 6).

Du et al. first introduced the L4 sagittal vertical axis (L4-SVA), defined as the distance between the L4 plumb line and the posterosuperior corner of S1, as a parameter representing the anterior shift of the loading axis of the lower lumbar spine [9]. Kitaori et al. considered L4-SVA as a predictive parameter for this rare complication, as their case deteriorated to 64 mm after initial surgery [5]. Although the normal value of L4-SVA has not been clearly defined in the literature, in our two cases, preoperative L4-SVA measured 68 mm and 64 mm, respectively, and postoperatively, they were 62 mm and 65 mm, closely approximating their reported values both before and after surgery. Unfortunately, complete postoperative standing radiographs could not be obtained due to the immediate onset of the fractures.

In considering strategies to prevent pedicle fractures, preoperatively, bone quality assessment using a DXA scan or CT and bone anabolic therapy for quality improvement should be considered essential [10]. Surgically, while cement-augmented screws have been proposed as a reinforcement strategy, their effectiveness has been reported to be limited [4,7]. Postoperatively, Wanivenhaus et al. proposed rehabilitation protocols incorporating early sitting restrictions, although the efficacy of this approach remains unverified [7]. Generally, the fracture frequently necessitates the extension of fusion to the pelvis [3-7]. However, our cases represent the first report of successful conservative management using bed rest combined with teriparatide, avoiding the need for revision surgery. Both patients currently maintain pain-free daily activities without requiring additional surgery. Teriparatide has been shown to enhance both fusion rates and bone strength in PLIF procedures [11-13]. However, we attribute the success of this treatment largely to early intervention when fracture displacement was minimal. The fracture has been reported to occur early, typically within one month postoperatively [5,7], which was consistent with our cases occurring at 5 and 36 days after surgery. Based on these observations, we suggest that careful monitoring in the early postoperative period, particularly in cases with high postoperative L4-SVA values, is crucial for the early detection and successful conservative treatment of these fractures.

## Conclusions

We report two cases of bilateral L5 pedicle fractures following L4-5 single-level PLIF that were successfully managed through conservative treatment. These fractures typically necessitate extending the fusion construct to either the sacrum or ilium. Our experience demonstrates that early detection, particularly in female patients with suboptimal sagittal alignment parameters, such as high L4-SVA values, is crucial, as these fractures predominantly occur within the first postoperative month. When identified before significant displacement occurs, conservative treatment combining bed rest with teriparatide administration may effectively achieve bone union, potentially sparing patients from extensive revision surgeries and associated morbidity.

## References

[1] Okuda S, Miyauchi A, Oda T, Haku T, Yamamoto T, Iwasaki M (2006). Surgical complications of posterior lumbar interbody fusion with total facetectomy in 251 patients. J Neurosurg Spine.

[2] Ha KY, Kim YH (2003). Bilateral pedicle stress fracture after instrumented posterolateral lumbar fusion. A case report. Spine (Phila Pa 1976).

[3] Jorge JP, Carvalho N (2019). Adjacent bi-level bilateral pedicle stress fractures after instrumented posterolateral lumbar fusion-a case report and review of the literature. Eur J Orthop Surg Traumatol.

[4] Kim HS, Ha SW, Ju CI, Kim SW (2017). Adjacent bilateral stress pedicle fractures after instrumented lumbar fusion: a case report. Korean J Neurotrauma.

[5] Kitaori T, Ota M, Tamura J (2023). Bilateral L5 pedicle fracture with L5-S1 spondylolisthesis after single-level L4-5 posterior lumbar interbody fusion: illustrative case. J Neurosurg Case Lessons.

[6] Lattig F, Fekete TF, Jeszenszky D (2010). Management of fractures of the pedicle after instrumentation with transpedicular screws. A report of three patients. J Bone Joint Surg Br.

[7] Wanivenhaus F, Bauer DE, Laux C (2022). Risk factors for L5 pedicle fractures after single-level posterior spinal fusion. Spine J.

[8] Roussouly P, Gollogly S, Berthonnaud E, Labelle H, Weidenbaum M (2006). Sagittal alignment of the spine and pelvis in the presence of L5-s1 isthmic lysis and low-grade spondylolisthesis. Spine (Phila Pa 1976).

[9] Du CZ, Li S, Xu L, Zhou QS, Zhu ZZ, Sun X, Qiu Y (2019). Sagittal reconstruction of lumbosacral contiguous double-level spondylolytic spondylolisthesis: a comparison of double-level and single-level transforaminal lumbar interbody fusion. J Orthop Surg Res.

[10] Sardar ZM, Coury JR, Cerpa M (2022). Best practice guidelines for assessment and management of osteoporosis in adult patients undergoing elective spinal reconstruction. Spine (Phila Pa 1976).

[11] Cho PG, Ji GY, Shin DA, Ha Y, Yoon DH, Kim KN (2017). An effect comparison of teriparatide and bisphosphonate on posterior lumbar interbody fusion in patients with osteoporosis: a prospective cohort study and preliminary data. Eur Spine J.

[12] Ebata S, Takahashi J, Hasegawa T (2017). Role of weekly teriparatide administration in osseous Union enhancement within six months after posterior or transforaminal lumbar interbody fusion for osteoporosis-associated lumbar degenerative disorders: a multicenter, prospective randomized study. J Bone Joint Surg Am.

[13] Miyazaki M, Ishihara T, Abe T, Kanezaki S, Hirakawa M, Iwasaki T, Tsumura H (2022). Analysis of treatment effect with teriparatide on device-related vertebral osteopenia after lumbar spinal interbody fusion using Hounsfield unit values: a retrospective cohort study. Medicine (Baltimore).

